# A Review of Web-Based Nutrition Information in Spanish for Cancer Patients and Survivors

**DOI:** 10.3390/nu14071441

**Published:** 2022-03-30

**Authors:** Fjorida Llaha, Alba Ribalta, Lorena Arribas, Marta Bellver, Elena Roura, Núria Guillén-Rey, Isabel Megias-Rangil, Clara Alegret-Basora, Anna Tresserra-Rimbau, Raul Zamora-Ros

**Affiliations:** 1 Unit of Nutrition and Cancer, Cancer Epidemiology Research Program, Catalan Institute of Oncology (ICO), Bellvitge Biomedical Research Institute (IDIBELL), 08908 Barcelona, Spain; fllaha@idibell.cat (F.L.); albaribaltac@gmail.com (A.R.); 2 Clinical Nutrition Unit, Catalan Institute of Oncology (ICO), 08908 Barcelona, Spain; larribas@icooncologia.net (L.A.); marta.bellver@iconcologia.net (M.B.); 3 Alicia Foundation, 08272 Sant Fruitós de Bages, Spain; elena@alicia.cat; 4 Unit of Nutrition, Sant Joan de Reus University Hospital, 43204 Reus, Spain; nuria.guillen@salutsantjoan.cat (N.G.-R.); isabelclara.megias@salutsantjoan.cat (I.M.-R.); clara.alegret@salutsantjoan.cat (C.A.-B.); 5 Department of Nutrition, Food Sciences and Gastronomy, School of Pharmacy and Food Sciences, University of Barcelona, 08028 Barcelona, Spain; annatresserra@ub.edu; 6 CIBER Physiopathology of Obesity and Nutrition (CIBEROBN), Institute of Health Carlos III, 28029 Madrid, Spain; 7 Nutrition and Food Safety Research Institute (INSA-UB), University of Barcelona, 08921 Santa Coloma de Gramenet, Spain

**Keywords:** nutrition, cancer patients, cancer treatment, dietary guidelines, diet, health information, websites

## Abstract

Nutrition education resources are of interest for cancer patients and survivors throughout the cancer continuum. We examined the web-based nutrition information in Spanish for cancer patients and survivors provided by national cancer organizations (NCOs). The Guide to Internet Resources for Cancer and the membership list of the Union for International Cancer Control were searched to identify the NCOs. The International Patients Decisions Aid Standards (IPDAS) was used to describe the quality of the available information. We identified 20 NCOs that provided nutrition information aimed at a general audience on their websites. Web-based resources of nine NCOs were selected for presentation in this review. Website scores ranged between 20 and 24 in the IPDAS scale (maximum score = 31). The selected NCOs offered reliable and safe information. Healthy eating information for cancer patients and management of side-effects was provided by all websites. Information was more limited for cancer survivors. We recommend that NCOs increase the possibilities for personalized recommendations and interaction with the content by including instrumental tools on their websites.

## 1. Introduction

Cancer is one of the most frequent causes of morbidity and mortality worldwide, and 19.3 million incident cases and almost 10.0 million deaths were estimated for 2020 [1]. Cancer and its treatment cause metabolic and nutritional derangements with a subsequent increased risk of malnutrition and muscle mass loss in cancer patients [2,3,4,5]. The prevalence of malnutrition ranges from 15–40% at the time of cancer diagnosis and can increase up to 80% in cases with advanced tumors [6]. Malnutrition and muscle mass loss negatively impact on patients’ outcomes, decreasing survival and ability to complete treatment and increasing post-surgical complications and public health system costs [7,8,9]. Common causes of poor nutrition intake during cancer treatment are anorexia, xerostomia, early satiety, nausea, vomiting, dysgeusia, dysphagia, depression, anxiety, and pain [10]. These symptoms can be due to the cancer itself and/or the side effects of cancer treatments (surgery, chemotherapy, and radiotherapy), which are usually very aggressive [11]. Changes in the metabolism of proteins, carbohydrates, and fats are major determinants of weight loss. Cachexia has been often recognized as an adverse effect of cancer. Cancer cachexia is a multifactorial syndrome, characterized by ongoing loss of skeletal muscle mass, reduced food intake, and abnormal metabolism, producing a negative balance of protein and energy intake [12]. To prevent cancer cachexia development, patients need regular assessment of their nutritional status, advice on practical methods to ensure adequate nutrition, and interventions that enhance nutrition. Increased energy and protein intake are often needed during cancer treatment. Lifestyle interventions (diet and exercise) have shown a positive effect in clinical outcomes by improving physical function, body composition, fatigue, quality of life, and even survival in cancer patients [13,14,15]. However, weight loss and malnutrition in patients with cancer is not always assessed or managed actively [16,17]. An increase of body weight can also occur in some patients with cancer, for example, in patients with breast and prostate cancer due to hormonal treatments [18,19]. There is evidence linking obesity with lower survival, certainly observed among women with breast cancer [20]. The heterogeneity of cancer’s adverse effects demonstrates the need for specific dietary guidelines, considering the cancer type and treatment plan. Cancer survivors remain at risk of cancer recurrence and have a higher risk for diabetes and cardiovascular disease [21,22]. A healthy diet, often combined with physical activity, can improve prognosis in cancer survivors [23]. However, their adherence to dietary and physical activity recommendations is relatively low [24]. Principal reasons for ineffective nutritional practice among cancer patients and survivors include lack of knowledge and time of the cancer care team [16,25]. Additional nutrition and lifestyle education resources are, therefore, of interest for cancer patients and survivors throughout the cancer continuum.

Digital and online information empowers citizens to make their own health decisions, including diet choices and cancer management [26,27]. Online health resources are used for four primary reasons: (i) to gain knowledge about the diagnosed disease, (ii) to obtain advice from other patients with the same disease, (iii) to receive social support, and (iv) to communicate with health professionals [28]. Unfortunately, a significant amount of online medical information is not peer-reviewed by experts and may contain inaccurate or misleading information [29]. Despite that, online resources have many potential benefits, such as improving patients’ cancer-related knowledge [30], self-help skills, and psychological outcomes [31,32]. Internet users for cancer information do not value all of the information equally [33]. For instance, findings from previous studies revealed that cancer patients favor reliable and credible resources, especially those using and discussing information from websites developed by well-known cancer organizations, medical centers, universities, and the government [34,35]. Thus, the aim of our review was to assess the availability and quality of the web-based nutrition information in Spanish for cancer patients and survivors provided by the national cancer organizations (NCOs).

## 2. Materials and Methods

Our review assessed the availability and the quality of the web-based nutrition information for cancer patients and survivors in Spanish from NCOs. We followed a systematic review methodology to reduce the search bias.

### 2.1. Search Strategy and Data Extraction 

In September 2021, we searched the Guide to Internet Resources for Cancer (www.cancerindex.org accessed on 15 September 2021) and the membership list of the Union for International Cancer Control (UICC, Geneva, Switzerland, www.uicc.org accessed on 15 September 2021) to identify NCOs with relevant content in Spanish. In both databases, we checked the membership list of Spanish-speaking countries and the US. Organizations from the US were also considered due to their high Hispanic and Latino American population [36]. In addition, we looked at the websites of the ministries of health of Spanish-speaking countries, and we contacted 31 NCOs from Latin America for more relevant information. Non-defunct links to other suggested or collaborative cancer organizations provided on the identified NCO websites were also considered for screening. Links to nutrition-cancer-related content from organizations without cancer management as the main mission were not included. Those aiming to provide nutrition information for cancer patients and survivors and which were freely accessible were included. For the present review, we extracted data regarding nutrition content and format, interactive elements used on the site, country of origin, languages, and social media linkage. If the NCOs provided different websites, we considered data only from the websites aimed at cancer patients and survivors. We did not extract information about other health recommendations, such as physical activity, artificial feeding, psychological support, smoking, vaccination, early detection/screening, sun exposure, or environmental pollutants. The data extraction was carried out in October 2021. Two authors (Fjorida Llaha (F.L.) and Alba Ribalta (A.R.)) independently completed the search and data extraction process. Disagreements were discussed between all authors until a consensus was reached.

### 2.2. Quality Assessment and Websites Navigation

The quality of the nutrition content was determined using the adapted validated International Patients Decisions Aid Standards (IPDAS) scale [37], used also in similar work [38]. The IPDAS has eight items and 31 sub-items. We adapted the scoring scheme by awarding 1 point to each sub-item if the material partially or completely fulfilled the quality criteria and 0 if the criteria was not met in any way. Thus, the maximum score was 31. A detailed assessment of nutrition content quality using the IPDAS is given in Supplementary Materials Table S1. We referred to the Word Cancer Research Fund/American Institute for Cancer Research (WCRF/AICR) recommendations for cancer survivors [39] and the European Society for Clinical Nutrition and Metabolism (ESPEN) [40] nutrition and cancer guidelines as the gold standard for the assessment of the nutrition information quality. Only the content in Spanish was evaluated. Social media linkage was considered to explore the expansion of the content beyond the website. However, the content provided through channels beyond the websites, such as social media or in-person events, was not assessed. Two authors (F.L. and A.R.) independently completed the IPDAS scale. Disagreements were discussed between all authors until a consensus was reached.

## 3. Results

### 3.1. National Cancer Organization (NCO) Websites

From our search, we identified 100 NCOs that provided content in Spanish on their websites. Twenty out of the 100 NCOs contained nutrition-cancer-related information addressed to the general audience (Supplementary Materials Table S2). Only the website of the Spanish Ministry of Health, Consumer Affairs, and Social Welfare contained links to NCOs. The web-based resources of nine NCOs with major nutrition content for cancer patients and survivors were selected for presentation in this review (Table 1). Six NCOs were originally from Spain, two from the US, and one from Venezuela (Table 2). The primary affiliation was non-profit organizations, except one US-government organization (NCI, Table 1). Few Spanish websites presented content in other co-official languages spoken in Spain: three in Catalan and one in Basque (Table 2). The newest launched websites included in this review (www.menjardurantelcancer.cat accessed on 30 October 2021) were found and developed by members of our research group.

### 3.2. Nutrition Information

Similar to the recommendations of the WCRF/AICR for cancer survivors [39], websites advised maintaining a healthy body weight and following well-balanced diets. They recommended decreasing the intake of high-fat processed foods, refined starches, sugars, and red and processed meat; avoiding alcohol intake; and increasing the intake of wholegrains, legumes, fruits, and vegetables. These recommendations are similar to those given for cancer prevention [41]. Nutrition information for cancer patients was mostly focused on the adequate intake of nutrients and energy and on the management of the potential side effects of cancer and its treatments. Guidelines for the side-effect management were consistent throughout the websites. For example, a diet rich in fiber was recommended for constipation, while a low-fiber diet was recommended for diarrhea. Content at pre- and post-treatment was more limited. At pre-treatment, websites advised to eat healthily and maintain a similar body weight as before the treatment and to be aware and discuss the management of possible side effects of cancer treatment with the cancer care team. Patients were recommended to (i) buy larger amounts of their favorite foods (thus, they would not need to go shopping very often); (ii) cook in advance and refrigerate foods in meal-sized portions; and (iii) talk to a friend or relative to help with shopping and cooking. Visiting the dentist to ensure optimal oral health before treatment was also highlighted. At post-treatment, patients were advised to first discuss any food and dietary restrictions with the cancer care team and to follow a healthy well-balanced diet; to eat a variety of fruits and vegetables every day, high-fiber foods, and low-fat foods; to limit the intake of red and processed meat; to avoid alcohol drinks; and for patients that were overweight/with obesity, to consider losing weight by reducing calorie intake and increasing physical activity. Three NCOs (Asociación Española Contra el Cáncer (AECC,) American Cancer Society (ACS), and Sociedad Anticancerosa de Venezuela (SAV); Table 1) had separate sections for children, offering menus and recipes. Two websites (Asociación Española de Afectados por Linfoma, Mieloma, Leucemia (AEAL) and Federació Catalana Entitats Contra el Càncer (FECEC); Table 1) gave an overview about malnutrition during cancer and the methods for assessing nutritional status in pre-treatment. Patients were recommended to require professional assistance if they experienced involuntary weight loss, loss of appetite, felt tired, and had difficulties performing physical exercise. Together with our website (www.menjardurantelcancer.cat accessed on 30 October 2021), two others (AECC, FECEC, Table 1) shared content that helped to dispel nutrition-cancer related myths. The most-common myths were regarding red meat, sugar, dairy products, soya, gluten, anti-cancer foods, anti-cancer diets, anti-cancer supplements, kitchen utensils and cooking methods, and the role of organic and conventional products in cancer. Two websites (FECEC, Sociedad Española de Oncología Médica (SEOM), Table 1) contained examples of foods and medicinal plants that could interact with cancer treatments, such as Hipericum perforatum, grapefruit, and soya products. Extensive content regarding food safety was offered by four NCOs (ACS, AEAL, National Cancer Institute (NCI), and SAV; Table 1), which emphasized recommendations for food handling, cross-contamination, grocery shopping, and eating outside.

### 3.3. Quality Assessment and Website Navigation

The promotion of nutrition information for a general audience was not the only objective of the selected NCOs. They also aimed to support investigation in cancer and to promote educational materials for health professionals. Despite the broad objectives, the nutrition content addressed to patients was easily accessible on their websites. In general, the selected websites were well-organized, with headings, subheadings, internal links, and an internal search engine. We observed that 2–4 clicks were needed to reach the nutrition-cancer content from the homepage. To interact with the audience (contact or receive feedback), the selected websites mostly provided a phone number or an email (Table 2). All identified websites were linked to at least two social media platforms, mostly to Twitter, Facebook, and Instagram. Five of the websites had the highest number of followers on Facebook (AECC, Geicam, Investigación en Cáncer de Mama (GEICAM), AEAL, ACS, and NCI; Table 2), two on Twitter (SEOM and FECEC; Table 2), and one on Instagram (SAV, Table 2). Our website (www.menjardurantelcancer.cat accessed on 30 October 2021) is not linked to social media platforms; however, its content has been included on the social media platforms of the founder organizations.

The IPDAS score of the selected websites is summarized in Table 3. IPDAS scores ranged from 20 to 24, with a mean score of 22.9 (1.9). All websites started with a clear mission statement and were awarded maximum points (3 points) as they clearly described their purposes and missions. Eight of the websites were awarded 5 and 6 points for item 2 as they correctly described the health condition, listed and described treatments or lifestyle options for cancer management and their benefits, reported the side effects of treatments, and described uncertainty concerning current evidence. Except for our website (www.menjardurantelcancer.cat accessed on 30 October 2021), all others were awarded one point for informing the audience about rates of cancer incidence (item 3). Information accuracy (item 4, maximum points = 4) among all websites showed a mean score of 2.3 (0.5). However, we consider the available information on all websites as accurate, as it was in line with the predefined gold standard evidence [39,40]. Although all websites clearly stated authorship and credentials for information compilation, sources of evidence were not always referenced. Websites were awarded a mean score 3.4 (0.5) for item 5. We considered the available information helpful for patients to make appropriate decisions, suggesting practical content, such as menus and recipes. Websites were evaluated highly for item 6 and 7, as they included authors’/developers’ credentials or qualifications, and the content was presented with reading aids such as an index, bullet points, summaries, pictures, and tables and was consistent in design and layout. The mean score for item 8 was 1, since not all websites reported the date of content publication or included sources of further information.

## 4. Discussion

In this review, we assessed the availability and the quality of nutrition information for cancer patients and survivors in Spanish provided by NCOs. The available information was accurate and in line with the most updated scientific evidence. Healthy eating information and content focused on the management of side effects during cancer treatments was provided by all websites. This is an important finding, as the management of side effects, food, and nutrition are among the greatest information needs of cancer patients [42,43]. Content for cancer survivors was more limited but well addressed for cancer prevention.

One of the websites (GEICAM, Table 1) was specific for patients with breast cancer and another (AEAL, Table 1) for patients with lymphoma, myeloma, and leukemia. Other NCO websites (SEOM, FECEC, and ACS; Table 1) structured or divided the information by tumor site, such as colorectal, prostate, pancreas, kidney, lung, and breast cancer. Although all websites addressed the most-frequent side effects that appear during cancer, presenting the content according to tumor site could assist in the selection of information provided to patients with specific cancers. Previous findings suggest that patients may reject high quality information if it does not appear to be aimed at people with the same condition [44]. Moreover, different degrees of side effects can appear, and specific nutrition recommendations cannot be applied to different cancer types. For example, a significant nutritional deterioration and a decrease in body weight and body mass index (BMI) can occur in head and neck cancer patients, who can easily become moderately to severely malnourished [45], while an increase in body weight can occur in patients with breast and prostate cancer [18,19]. Among the websites, we observed that the information at pre-treatment was limited and not always addressed. Improvements in nutritional support prior to treatment reduce the incidence of infections and length of hospital stay [46]. Moreover, an early nutritional assessment with dietetic counseling prior to the treatment of patients with a high prevalence of malnutrition, such as head and neck tumors, seems to be more effective [45]. Common advice of dietary guidelines during cancer treatment was to ensure an adequate calorie and protein intake. Most of the websites offered practical strategies on how to prepare high protein and high calorie meals. To help patients understand this approach, nutrition information, including protein and calorie amounts, was provided for each recipe on our website (www.menjardurantelcancer.cat accessed on 30 October 2021). We identified a lack of details regarding high protein or high calorie intake to make specific dietary recommendations, and there was no guidance on the long-term consumption of high protein or high calorie foods. The same gap was also identified by authors that explored information available in English-speaking countries [38,47]. However, counseling with a dietitian or healthcare professional was always recommended for personalized planning care.

The quality of the online health information depends not only on its veracity, but also on features associated with its presentation that generate trust [48]. There is a consensus that a clear layout, interactive features, and author credentials have a positive effect on confidence and credibility, whereas the presence of commercial advertisements has a negative effect [48]. Among the selected websites for this review, we observed that the use of presentation features such as the date of content publication, sources of evidence, or links to further/similar information was not consistent. Links to further or similar information offered on other websites might be worth including, as patients looking for online information in the health domain are more likely to trust websites that provide content that they can verify themselves [49]. Advertising rarely appeared among the websites presented in this review. The use of visual aids improves the health literacy outcome [50]. Videos are the most effective audio-visual aids [50]. All websites presented in this review used frequent visual aids, such as tables and pictures; however, videos were rarely used, except for our website (www.menjardurantelcancer.cat accessed on 30 October 2021), where all content was also presented in a video format. Only one website (AEAL, Table 1) linked to a forum website to interact with the audience. Nevertheless, the forum website was not updated, and the disposal of a professional moderator monitoring user interaction was not stated. Such elements might be of interest to include, as for cancer patients, connecting with others in online communities provides not only information but also emotional support, reducing the feeling of stress and uncertainty [51,52]. In a previous study with cancer patients, the use of instrumental tools was considered the most valuable online activity [53]. Only one of the selected websites (ACS, Table 1) provided quizzes; a calculator for BMI, calories, exercise activity, and heart rate; and a cancer risk assessment tool (the DEFENDER, https://thedefender.cancer.org/ accessed on 5 October 2021). The DEFENDER gives personalized recommendations for cancer prevention based on personal variables, such as age; gender; race; height; weight; smoking; alcohol intake; sitting hours; moderate and vigorous physical activity hours; fruit, vegetables, and meat consumption; sun protection habits; and family history of prostate, breast, and colorectal cancer. Unfortunately, this instrument was not available in Spanish.

Similar studies for English-speaking countries [38,47] have suggested that cancer websites should consider a specific online platform to provide more focused information on nutrition, due to the great variety of nutritional needs during cancer. In this approach, our website (www.menjardurantelcancer.cat accessed on 30 October 2021) presents an important step forward to bridge this gap. A limitation of our website development (www.menjardurantelcancer.cat accessed on 30 October 2021) was not, as yet, considering patients’ opinions to assess the utility and the usability of the website, as on most of the other websites. To construct an outline of the websites and nutrition information quality, we used the validated IPDAS scale. The IPDAS was completed by members of our research group, and the scoring might differ from the patients’ points of view. In addition, the screening of the available information was limited to a fixed time period (October 2021) and did not present the dynamic changes that were undergone on the websites.

## 5. Conclusions

Healthy eating information and content focused on the management of side effects during cancer treatments was provided by all websites. Nutrition guidelines for cancer survivors were not always addressed but were well-described for cancer prevention. The possibilities for personalized guidelines and interaction with web-based information remain uncovered. Furthermore, practical strategies for vegan, vegetarian, and coeliac cancer patients and survivors were missing. Perhaps, website and app developers in Spanish-speaking countries or beyond should consider the inclusion of instrumental tools for personalized nutrition-cancer guidelines. Besides common variables used in nutrition apps, such as age, gender, patients’ food preferences, height, weight, and physical activity, we also recommend considering cancer type, disease phase, treatment plan, and side effects. We also recommend to the health-related website developers to consistently report the content date of publication, to report more in-depth evidence-sources, and to make it easier to verify the author credentials of the reported content in order to increase the quality perception by the general audience.

The close collaboration among a multidisciplinary team to establish an individual nutritional intervention for cancer patients is the best practice. When the personalized nutritional care is uncovered in the daily clinical practice, the available web-based nutrition information provided by NCOs included in this review can be used by health practitioners as suggestive, reliable, and safe information resources to their patients. This might be especially of interest for older adult patients, as they are less likely to feel confident evaluating healthy resources on the internet [54]. Moreover, the content of these websites is easily accessible and consumable (videos), especially in the case of our website (www.menjardurantelcancer.cat accessed on 30 October 2021). However, to provide more specific dietary recommendations, further research is needed to better define nutrition guidelines in oncology [55].

## Figures and Tables

**Table 1:** Nutrition content in the selected websites.

Organizations/ Websites	Disease Phase	Nutrition Content	Side Effects
AECC www.aecc.es/es accessed on 5 October 2021	Prevention, during treatment	Healthy eating in adults and children, side effects, cancer myths, recipes	Anorexia, mucositis, dysgeusia, diarrhea, constipation, dysphagia, lactose intolerance, nausea, and vomiting
GEICAM www.geicam.org/ accessed on 5 October 2021	Prevention, during and post treatment	Healthy eating, side effects, recipes	Anorexia, mucositis, dysgeusia, diarrhea, constipation, dysphagia, nausea and vomiting, xerostomia, fatigue, weight loss, weight gain
AEAL www.aeal.es/ accessed on 5 October 2021	During and post treatment	Healthy eating, side effects, food safety, malnutrition, and nutritional status assessment	Anorexia, mucositis, dysgeusia, diarrhea, constipation, dysphagia, nausea, and vomiting
SEOM/Oncosaludable ^1^ www.oncosaludable.es/ accessed on 5 October 2021	Prevention and during treatment	Healthy eating, side effects, nutrient-drug interaction, menus, recipes	Anorexia, mucositis, xerostomia, esophagitis, dysgeusia, diarrhea, constipation, nausea and vomiting, low defenses, weight loss
FECEC www.juntscontraelcancer.cat accessed on 5 October 2021	Pre, during, and post treatment	Healthy eating, side effects, nutrient-drug interactions, malnutrition and nutritional status assessment, cancer myths, menus, recipes	Anorexia, mucositis, dysgeusia, diarrhea, constipation, dysphagia, nausea and vomiting, xerostomia, mouth ulcers, early satiety, high blood pressure, diabetes, pancreatic insufficiency, weight loss, weight gain, liquid retention, ileostomy, colostomy, intestinal obstruction
ICO/Què i Com Menjar Durant el Càncer ^1^, www.menjardurantelcancer.cat accessed on 30 October 2021	During treatment, survivorship	Healthy eating in adults, side effects, cancer myths, recipes	Anorexia, constipation, diarrhea, nausea and vomiting, mucositis, dysgeusia, dysphagia to solid foods, dysphagia to liquid foods
SAV/Ayuda al Paciente Oncológico ^1^, www.ayudaalpacienteoncologico.org.ve/, La Lonchera de mi Hijo ^1^ www.laloncherademihijo.org/ accessed on 5 October 2021	Prevention, pre, during, and post treatment	Healthy eating in adults and children, food safety, food portions, food groups, side effects, menus, recipes	Anorexia, mucositis, xerostomia dysgeusia, diarrhea, constipation, dysphagia, nausea and vomiting, weight gain
ACS www.cancer.org/es/ accessed on 5 October 2021	Prevention; pre, during, and post treatment; survivorship	Healthy eating in adults and children, side effects, food safety, meal planification, recipes	Anorexia, dysgeusia, diarrhea, constipation, dysphagia, nausea and vomiting, xerostomia, mouth ulcers, weight gain, fatigue
NCI www.cancer.gov/espanol accessed on 5 October 2021	Prevention, pre, during, and post treatment, survivorship	Healthy eating in adults, side effects, food safety, meal planification, recipes	Anorexia, mucositis, dysgeusia, diarrhea, constipation, dysphagia, nausea and vomiting, xerostomia, esophagitis, lactose intolerance, weight gain, weight loss

^1^ Separate websites of the organizations with information aimed to a general audience. ACS, American Cancer Society; AEAL, Asociación Española de Afectados por Linfoma, Mieloma, Leucemia; AECC, Asociación Española Contra el Cáncer; FECEC, Federació Catalana Entitats Contra el Càncer; GEICAM, Geicam, Investigación en Cáncer de Mama; ICO, Catalan Instiute of Oncology; NCI, National Cancer Institute; SAV, Sociedad Anticancerosa de Venezuela; SEOM, Sociedad Española de Oncología Médica.

**Table 2:** Other features of the selected websites.

Organizations/Websites	Country of Origin	Language	Content Format	Social Media Platforms	Interaction Methods
AECC www.aecc.es/es accessed on 5 October 2021	Spain	Spanish, Catalan ^1^, Basque ^2^	Webpage (Video-recipes), PDF	Facebook ^3^, Twitter Instagram, YouTube	Email, phone, social share buttons
GEICAM www.geicam.org/ accessed on 5 October 2021	Spain	Spanish, English	Webpage, PDF	Facebook ^3^, Twitter, Instagram, YouTube, LinkedIn	Email, phone, social share buttons
AEAL www.aeal.es/ accessed on 5 October 2021	Spain	Spanish	Webpage,	Facebook ^3^, Twitter	Email, phone, forum website
SEOM/Oncosaludable ^4^ www.oncosaludable.es/ accessed on 5 October 2021	Spain	Spanish	Webpage, PDF		Email, phone
FECEC www.juntscontraelcancer.cat accessed on 5 October 2021	Spain	Spanish, Catalan ^1^, English	PDF	Facebook, Twitter ^3^, Instagram, YouTube	Email, phone, social share buttons, comments box
ICO/Què i Com Menjar Durant el Càncer ^4^, www.menjardurantelcancer.cat accessed on 30 October 2021	Spain	Spanish, Catalan ^1^, English	Webpage (Video-recipes), Podcast, PDF		Social share buttons
SAV/Ayuda al Paciente Oncológico ^4^, www.ayudaalpacienteoncologico.org.ve/ La Lonchera de mi Hijo ^4^, www.laloncherademihijo.org/ accessed on 5 October 2021	Venezuela	Spanish	Webpage, PDF	Facebook, Twitter, Instagram ^3^	Email, phone, forum on WhatsApp
ACS www.cancer.org/es/ accessed on 5 October 2021	US	Spanish, English, Others ^5^	Webpage (Videos), PDF	Facebook ^3^, Twitter, Instagram	Phone, live chat, comments box
NCI www.cancer.gov/espanol accessed on 5 October 2021	US	Spanish, English	Webpage, PDF	Facebook ^3^, Twitter, Instagram, LinkedIn	Phone, live chat

ACS, American Cancer Society; AEAL, Asociación Española de Afectados por Linfoma, Mieloma, Leucemia; AECC, Asociación Española Contra el Cáncer; FECEC, Federació Catalana Entitats Contra el Càncer; GEICAM, Geicam, Investigación en Cáncer de Mama; ICO, Catalan Institute of Oncology; NCI, National Cancer Institute; SAV, Sociedad Anticancerosa de Venezuela; SEOM, Sociedad Española de Oncología Médica; PDF, Portable Document Format. ^1^ A co-official language of an autonomous region in eastern Spain, Catalonia; ^2^ A co-official language of an autonomous region in northern Spain, Basque Countries; ^3^ Platform with the highest number of followers; ^4^ Separate websites of the organizations that provide educational material aimed at patients and/or relatives for better management of cancer; ^5^ Arabic, Chinese, French, Haitian Creole, Hindi, Korean, Polish, Portuguese, Russian, Tagalog, Vietnamese.

**Table 3:** International Patients Decisions Aid Standards Scores (IPDAS).

Scores for Item and Total Scores	AECC	GEICAM	AEAL	SEOM/Oncosaludable	FECEC	SAV/Ayuda al Paciente Oncológico	ACS	NCI	ICO/Què i Com Menjar Durant El Càncer	Mean (SD)
1. Starts with a clear statement of aims? (Max: 3)	3	3	3	3	3	3	3	3	3	3 (0.0)
2. Provides unbiased and detailed information about options? (Max: 7)	6	4	5	6	6	5	5	6	6	5.4 (0.7)
3. Presents probabilities of outcomes in an understandable way? (Max: 3)	1	1	1	1	1	1	1	1	0	0.9 (0.3)
4. Contains accurate information? (Max: 4)	2	3	2	3	2	3	2	2	2	2.3 (0.5)
5. Helps patients make appropriate decisions? (Max: 4)	4	3	3	3	4	3	4	4	3	3.4 (0.5)
6. Discloses conflicts of interest? (Max: 2)	2	2	1	2	2	2	2	2	1	1.8 (0.4)
7. Has a clear structure and layout? (Max: 6)	5	5	5	5	5	5	5	5	5	5 (0.0)
8. Helps the reader judge its reliability? (Max: 2)	1	1	0	2	1	1	1	2	0	1 (0.7)
Total score (Max: 31)	24	22	20	25	24	23	23	25	20	22.9 (1.9)

ASC, American Cancer Society; AEAL, Asociación Española de Afectados por Linfoma, Mieloma, Leucemia; AECC, Asociación Española Contra el Cáncer; FECEC, Federació Catalana Entitats Contra el Càncer; GEICAM, Geicam, Investigación en Cáncer de Mama; ICO, Catalan Institue of Oncology Max, Maximum points; NCI, National Cancer Institute; SD, Standard deviation; SAV, Sociedad Anticancerosa de Venezuela; SEOM, Sociedad Española de Oncología Médica.

## Data Availability

Data are available in the manuscript’s tables.

## Supplementary Materials

The following supporting information can be downloaded at https://www.mdpi.com/article/10.3390/nu14071441/s1. Table S1: International Patients Decisions Aid Standards Scores (IPDAS), Table S2: Cancer organizations in Spanish ordered by country.
