# Supplementary material for: A Review of Web-Based Nutrition Information in Spanish for Cancer Patients and Survivors
Source: Nutrients. 2022 Mar 30;14(7):1441. doi: 10.3390/nu14071441 (PMC9003392; doi:10.3390/nu14071441)
Supplement: Supplementary file 1 [file nutrients-14-01441-s001.zip › nutrients-1625874-supplementary.pdf]

Table S1. International Patients Decisions Aid Standards Scores (IPDAS).

	AECC	GEICAM	AEAL	SEOM / Oncosaludable	FECEC	SAV / Ayuda al Paciente Oncologico	ACS	NCI	ICO / Què i Com Menjar Durant el
**1. Start with a clear statement of aims?**									
1.1. Describes its purpose (e.g., to aid decision-making)	*	*	*	*	*	*	*	*	*
1.2. Describes what it covers (to help the reader judge whether it's worth carrying on)	*	*	*	*	*	*	*	*	*
1.3. Describes who it is for (i.e., which patient groups)	*	*	*	*	*	*	*	*	*
Points	3	3	3	3	3	3	3	3	3
**2. Provide unbiased and detailed information about options?**									
2.1. Describes the health condition	*	*	*	*	*	*	*	*	*
2.2. Describes the natural course without treatment									
2.3. Lists the treatment/management/lifestyle options	*	*	*	*	*	*	*	*	*
2.4. Describes benefits of options	*		*	*	*	*	*	*	*
2.5. Describes risks options (harms/side-effects/disadvantages)	*	*	*	*	*	*	*	*	*
2.6. Describes uncertainty around the current evidence (i.e., what is not known)	*			*	*		*		*
2.7. Describes procedures (i.e., treatments, targets, monitoring, behaviour change, etc.)	*	*	*	*	*	*	*	*	*
Points (out of 7)	6	4	5	6	6	5	6	5	6
**3. Present probabilities of outcomes in an understandable way?**									
3.1. Uses event rates specifying the population and, if appropriate, time period	*	*	*	*	*	*	*	*	
3.2. Compares outcome probabilities using the same numerator/denominator, time period, scale (i.e., if numerators/denominators, time periods or scales are used, they need to be consistent)									
3.3. Uses visual diagrams and/or places probabilities in context of other familiar events									
Points (out of 3)	1	1	1	1	1	1	1	1	0
**4. Contain accurate information?**									
4.1. Clearly states the evidence sources used in compiling the information		*		*				*	
4.2. Information quoted is in line with the most up-to-date clinical evidence	*	*	*	*	*	*	*	*	*
4.3. Where mentioned, prevalence estimates give an accurate impression of how common/rare the condition is									
4.4. Personal opinion and/or advertising are clearly distinguished from evidence-based information	*	*	*	*	*	*	*	*	*
Points (out of 4)	2	3	2	3	2	2	2	3	2
**5. Help patients to make appropriate decisions**									
5.1. Acknowledges (explicitly or implicitly) that the patient has decisions to make	*	*	*	*	*	*	*	*	*
5.2. Helps patients to imagine what it is like to live with the condition and/or treatment effects	*	*	*	*	*	*	*	*	*
5.3. Asks patients to consider factors (e.g., priorities, motivations, treatment outcomes) affecting possible courses of action	*				*	*	*		
5.4. Suggests ways and/or provides tools to help patients make decisions	*	*	*	*	*	*	*	*	*
Points (out of 4)	4	3	3	3	4	4	4	3	3
**6. Disclose conflicts of interest?**									
6.1. Includes authors' / developers' credentials or qualifications	*	*		*	*	*	*	*	*

6.2. Reports source of funding to develop and distribute the patient decision aid	*	*	*	*	*	*	*	*	
Points (out of 2)	2	2	1	2	2	2	2	2	1
**7. Have a clear structure and layout?**									
7.1. Is consistent in design and layout throughout	*	*	*	*	*	*	*	*	*
7.2. Includes aids to finding information (e.g., contents, index, site map, or search facility)	*	*	*	*		*	*	*	*
7.3. Important points are emphasised through the use of summaries and/or bullet points	*	*	*	*	*	*	*	*	*
7.4. Illustrates information with diagrams and/or pictures	*	*	*	*	*	*	*	*	*
7.5. Where diagrams appear, they are labelled and relate to the subject matter					*				
7.6. Sections are clearly separated	*	*	*	*	*	*	*	*	*
Points (out of 6)	5	5	5	5	5	5	5	5	5
**8. Help the reader judge its reliability**									
8.1. Reports date of publication	*	*		*	*	*	*		
8.2. Includes sources of further information				*			*	*	
Points (out of 2)	1	1	0	2	1	1	2	1	0
**Total points** (out of 31)	**24**	**22**	**20**	**22**	**24**	**23**	**25**	**23**	**20**

ASC, American Cancer Society; AEAL, Asociación Española de Afectados por Linfoma, Mieloma, Leucemia; AECC, Asociación Española Contra el Cáncer; FECEC, Federació Catalana Entitats Contra el Càncer; GEICAM, Geicam, Investigación en Cáncer de Mama; ICO, Institut Català d'Oncologia; Max, Maximum score; NCI, National Cancer Institute; SD, Standard deviation; SAV, Sociedad Anticancerosa de Venezuela; SEOM, Sociedad Española de Oncología Médica.

Table S2. National cancer organizations in Spanish ordered by country.

	Organizations	Country of Origin	Websites	Links to Nutrition-Cancer Content
1	Sociedad Latinoamericana y del Caribe de Oncología Médica (SLACOM)	Argentina	https://www.slacom.org/	
2	Instituto Nacional de Cáncer en Argentina	Argentina	https://www.argentina.gob.ar/	
3	Asociación Leucemia Mieloide de Argentina (ALMA)	Argentina	https://asociacionalma.org.ar/	
4	Fundación SALES	Argentina	https://sales.org.ar/	
5	Instituto Oncológico Henry Moore	Argentina	http://www.hmoore.com.ar/	http://www.hmoore.com.ar/pacientes.php
6	Liga Argentina de Lucha Contra el Cáncer	Argentina	https://www.lalcec.org.ar/	
7	Linfomas Argentina	Argentina	https://www.linfomasargentina.org/	
8	Fundación Argentina de Mieloma	Argentina	http://www.mieloma.org.ar/	
9	Fundación Pacientes Cáncer de Pulmón	Argentina	https://www.pacientescancerpulmon.org/	
10	Fundación ACIAPO	Argentina	https://fundacionaciapo.org.ar/	
11	Fundación FUCA Argentina	Argentina	https://fuca.org.ar/	
12	Movimiento Ayuda Cáncer de Mama (MACMA)	Argentina	http://www.macma.org.ar/	
13	Centro de Investigación, Educación y Servicio	Bolivia	http://www.cies.org.bo/	
14	Fundación Boliviana de Lucha Contra el Cáncer	Bolivia	https://www.fubolcancer.com/	
15	Fundación Arturo López Pérez	Chile	https://www.institutoncologicofalp.cl/	https://www.institutoncologicofalp.cl/apoyo-paciente/alimentacion-cancer/
16	Fundación Chile sin Cáncer	Chile	https://chilesincancer.cl/	
17	Instituto Nacional del Cáncer de Chile	Chile	https://www.incancer.cl/	
18	Fundación Chilena para el Desarrollo de la Oncología	Chile	http://www.cancerchile.cl/	
19	Corporación Nacional del Cáncer	Chile	https://www.conac.cl/	
20	Corporación Nacional Maxi-Vida	Chile	https://maxivida.cl/	
21	Instituto Nacional de Cancerología	Colombia	https://www.cancer.gov.co/	
22	Liga Colombiana Contra el Cáncer	Colombia	https://www.ligacancercolombia.org/	https://www.ligacancercolombia.org/galeria-multimedia/
23	Fundación Esperanza Viva	Colombia	https://fundacionesperanzaviva.com/	
24	Fundación Santa Fe de Bogotá	Colombia	http://www.fsfb.org.co	
25	Fundación SIMMON	Colombia	https://www.simmoncancer.org/	https://www.simmoncancer.org/docs/sobre-el-cancer/nutricion/
26	Asociación de Enfermería Oncológica Colombiana	Colombia	https://aeocol.com/	
27	Asociación de Enfermeras Oncohematológicas	Costa Rica	https://www.aeohpri.org/	

28	Fundación Amigos Contra el Cáncer Infantil	Dominican Republic	https://www.facci.org.do/	
29	Sociedad de Lucha Contra el Cáncer del Ecuador	Ecuador	https://www.solca.med.ec/	
30	CEPREME Juntos contra el cáncer	Ecuador	https://www.mujercepreme.com/	
31	Fundación Jóvenes Contra el Cáncer	Ecuador	https://jovenescontraelcancer.org/	
32	Liga Contra el Cáncer - Honduras	Honduras	https://www.ligacontraelcancer.hn/	
33	Federación Latinoamericana de Sociedades de Oncología	Latin America	https://www.flasca.com/	
34	Fundación Cima	Mexico	https://www.cimafundacion.org/	https://www.cimafundacion.org/guia-para-el-paciente-y-calidad-de-vida/
35	Asociación Mexicana de Sobrevivientes al Cáncer Oncoayuda	Mexico	https://oncoayuda.org/	
36	Cada de la Amistad para Niños con Cáncer	Mexico	https://www.casadelaamistad.org.mx/	
37	Grupo de Recuperación Total Reto	Mexico	https://www.gruporeto.org/	
38	Instituto Nacional de Cancerología	Mexico	http://www.incan-mexico.org/	
39	Sociedad Mexicana de Radioterapeutas	Mexico	https://www.somera.org.mx/	
40	Tómatelo a Pecho	Mexico	http://tomateloapecho.org.mx/	
41	Unidos, Asociación Pro-Trasplante de Medula Ósea. Francisco Casares Cortina	Mexico	https://www.uni2.org.mx/	
42	Asociación Mexicana de Lucha Contra el Cáncer	Mexico	https://www.amlcc.org/	
43	Fundación de Alba	Mexico	https://fundaciondealba.org/	
44	Asociación Mexicana de Leucemia y GIST	Mexico	http://ameleg.org.mx/	
45	Sociedad Mexicana de Oncología	Mexico	https://www.smeo.org.mx/	
46	Asociación Peruana Vida sin Cáncer	Peru	http://vidassincancer.org.pe/	
47	Esperantra Peru	Peru	http://www.esperantra.org/	
48	Fundación Peruana de Cáncer	Peru	https://fpc.pe/	
49	Instituto Peruano de Oncología & Radioterapia	Peru	https://www.ipor.pe/	https://www.ipor.pe/pacientes/nutricion-oncologica/
50	Sociedad Peruana de Oncología Medica	Peru	https://www.spomedica.org/	https://www.spomedica.org/informacion-para-pacientes/
51	Instituto Nacional de Enfermedades Neoplásicas de Perú	Peru	https://portal.inen.sld.pe/	
52	La Liga Peruana de Lucha Contra el Cáncer	Peru	https://ligacancer.org.pe/	
53	ALIADA Centro Oncológico Perú	Peru	https://www.aliada.com.pe/	
54	Asociación de Niños con Cáncer	Spain	https://afanoc.org/	
55	Asociación de Padres de Niños con Cáncer	Spain	https://www.asion.org/	
56	Fundación Instituto Valenciano de Oncología	Spain	https://www.ivo.es/	
57	Institut Català d'Oncologia	Spain	http://ico.gencat.cat/	https://menjardurantelcancer.cat/

				https://mejorsincancer.org/
58	Asociación Española de la Anemia de Fanconi	Spain	https://anemiadefanconi.org/	
59	El Grupo Español de Investigación en Cáncer de Ovario	Spain	https://www.geicogroup.com/	
60	Fundación Josep Carreras contra la Leucemia	Spain	https://www.fcarreras.org/es	https://www.fcarreras.org/es/alimentacion_1211484
61	Sociedad Española de Enfermería Oncológica	Spain	https://seeo.org/	
62	Federación Española de Cáncer de Mama	Spain	http://nuevofecma.vinagrero.es/	
63	Geicam, Investigación en Cáncer de Mama	Spain	https://www.geicam.org/	https://www.geicam.org/wp-content/uploads/2018/10/3251-MAIL-actualizacion-Guias-Nutricion-Ejercicio-Cancer-Mama.pdf
64	Asociación Española de Afectados por Linfoma, Mieloma y Leucemia	Spain	http://www.aeal.es/	http://www.aeal.es/alimentacion-y-nutricion/
65	Fundación CRIS Contra el Cáncer	Spain	https://criscancer.org/es/	
66	Fundación Theodora	Spain	https://es.theodora.org/	
67	Vall d'Hebron Institute of Oncology	Spain	https://www.vhio.net/	
68	Federació Catalana d'Entitats Contra el Càncer (FECEC)	Spain	https://www.juntscontraelcancer.cat/	https://www.juntscontraelcancer.cat/es/suport-serveis/viure-amb-cancer/
69	Sociedad Española de Oncología Medica	Spain	https://seom.org/<br>https://oncosaludable.es/	https://seom.org/informacion-sobre-el-cancer/prevencion-cancer<br>https://seom.org/seomcms/images/stories/recursos/Guias_Nutricion_Ejercicio_Cancer_Mama.pdf<br>https://seom.org/guia-actualizada-de-tratamientos/cuidados-de-soporte?start=5
70	Asociación Española Contra el Cáncer	Spain	https://www.aecc.es/es	https://www.contraelcancer.es/sites/default/files/ebooks/GuiaAlimentacionYCancer_2018_INTERACTIVO.pdf<br>https://www.contraelcancer.es/es/todo-sobre-cancer/prevencion/alimentacion<br>https://blog.contraelcancer.es/alimentacion-vida-sana/<br>https://blog.contraelcancer.es/consejos-para-pacientes-y-familiares/
71	El Grupo Español de Pacientes con Cáncer	Spain	http://www.gepac.es/	
72	Lymphomamieloma.com	Spain	https://www.linfomaymieloma.com/	
73	Asociación Andaluza de Hematología y Hemoterapia	Spain	https://www.aa-hh.org/	
74	Federación de Sociedades Españolas de Oncología (FESEO)	Spain	https://www.feseo.com/home?lang=es&setlang=es	
75	Sociedad Española de Hematología y Oncología Pediátricas (SEHOP)	Spain	https://www.sehop.org/	
76	Federación Española de Padres de Niños con Cáncer (FEPNC)	Spain	https://cancerinfantil.org/	
77	Asociación de Afectados por Cáncer de Pulmón	Spain	https://afectadoscancerdepulmon.com/	

78	Grupo Español de Cáncer de Pulmón	Spain	https://www.gecp.org/	
79	Societat Catalano-Balear d'Oncologia	Spain	http://webs.academia.cat/societats/oncologia/?p=page/html/activitats	
80	Centro de Investigación del Cáncer	Spain	https://www.cicancer.org/	
81	Centro Nacional de Investigaciones Oncológicas	Spain	https://www.cnio.es/	
82	Grup Oncològic Català Occità	Spain	http://www.grupgoco.org/index.php/es/	
83	Fundación Contra el Cáncer	Spain	https://www.fefoc.org/	
84	Fundación Porsaleu	Uruguay	https://porsaleu.org/	
85	Comisión Honoraria de Lucha Contra el Cáncer	Uruguay	https://www.comisioncancer.org.uy/	https://www.comisioncancer.org.uy/categoria/Alimentacion-saludable-24
86	American Cancer Society	US	https://www.cancer.org/es/	https://www.cancer.org/es/buscar.html?q=alimentacion https://www.cancer.org/content/dam/cancer-org/cancer-control/es/booklets-flyers/nutrition-for-the-patient-with-cancer-during-treatment-spanish.pdf
87	National Cancer Institute	US	https://www.cancer.gov/espanol	https://www.cancer.gov/espanol/cancer/sobrellevar/consejos-de-alimentacion.pdf
88	Cancer Care	US	https://www.cancercare.org/espanol	https://media.cancercare.org/publications/original/364-fs_nutrition_esp.pdf
89	Latinas Contra Cáncer	US	https://latinascontracancer.org/	https://latinascontracancer.org/health-education/
90	American Society of Clinical Oncology (ASCO)	US	https://www.cancer.net/es/	https://www.cancer.net/es/sobrevivencia/una-vida-saludable
91	Acoustic Neuroma Asociation	US	https://www.anausa.org/	
92	Cancer Research Institute	US	https://www.cancerresearch.org/	
93	Colon Cancer Alliance	US	https://www.ccalliance.org/espanol	
94	Lymphoma Research Foundation	US	https://lymphoma.org/es/	
95	Pancreatic Cancer Acrion Network	US	https://www.pancan.org/section_en_espanol/	
96	International Neuroendocrine Cancer Alliance	US	https://incalliance.org/	
97	Moffitt Cancer Centre	US	https://moffitt.org/	
98	International Waldenstrom Macroglobulinemia Foundation	US	https://iwmf.com/	
99	University of Miami Sylvester Comprehensive Cancer Center	US	https://umiamihealth.org/locations/sylvester-comprehensive-cancer-center	
100	Sociedad Anticancerosa de Venezuela	Venezuela	https://www.sociedadanticancerosa.org/	https://www.ayudaalpacienteoncologico.org.ve/ https://www.laloncherademihijo.org/

100 cancer organization with content in Spanish on their website. The organizations in green colour provided some information about nutrition. The organizations in yellow colour were selected for presentation in this review as they provided more extended content about nutrition for cancer management.
